# Cardiovascular risk assessment in patients with diabetes

**DOI:** 10.1186/s13098-017-0225-1

**Published:** 2017-04-20

**Authors:** Marcello Casaccia Bertoluci, Viviane Zorzanelli Rocha

**Affiliations:** 1 Departamento de Medicina Interna da Faculdade de Medicina da, Universidade Federal do Rio Grande do Sul, UFRGS, Rua Ramiro Barcelos 2400, Porto Alegre, ZIP 90035-003 RS Brazil; 2 Universidade Federal do Rio Grande do Sul, Programa de Pós-Graduação em Ciências Médicas, Rua Ramiro Barcelos 2400, Porto Alegre, 90035-003 Brazil; 3 Hospital de Clínicas de Porto Alegre- UFRGS, Serviço de Medicina Interna, Rua Ramiro Barcelos 2350 Sala 700, Porto Alegre, 90035-005 RS Brazil; 4 Lipid Clinic, Heart Institute (InCor) University of Sao Paulo, Sao Paulo, SP 05403-900 Brazil

**Keywords:** Cardiovascular disease, Risk stratification, Risk assessment, Type 2 diabetes, Risk factors

## Abstract

Although patients with diabetes have 2 to 4 times increased risk of cardiovascular morbidity and mortality than individuals without diabetes, recent studies indicate that a significant part of patients are in a lower cardiovascular risk category. Men younger than 35 years, women younger than 45 years, patients with diabetes duration of less than 10 years without other risk factors have a much lower risk than patients who have traditional cardiovascular risk factors, and subclinical or established coronary artery disease (CAD). These patients are not risk equivalent as stated in previous studies. On the contrary, when in the presence of traditional risk factors or evidence of subclinical coronary disease (e.g. high coronary calcium score), the coronary risk is much increased and patients may be classified at a higher-risk category. Recent guidelines do not anymore consider diabetes as a CAD risk equivalent and recommend cardiovascular risk stratification for primary prevention. Stratification of diabetic patients improves accuracy in prediction of subclinical CAD, silent ischemia and future cardiovascular events. Stratification also discriminates higher from lower risk patients who may need intensive statin or aspirin prevention, while avoiding overtreatment in lower risk cases. It may also allow the clinician to decide whether to intensify risk reduction actions through specific newer drugs for glucose control such as SGLT-2 inhibitors or GLP-1 agonists, which recently have shown additional cardiovascular protector effect. This review addresses the assessment of cardiovascular disease risk using traditional and non-traditional cardiovascular risk factors. It also reviews the use of risk calculators and new reclassification tools, focusing on the detection of subclinical atherosclerosis as well as silent ischemia in the asymptomatic patients with diabetes.

## Background

It is well known that type 2 diabetes (T2DM) is associated with increased cardiovascular morbidity and mortality [1]. Patients with T2DM have a two to fourfold increase in risk of incident coronary heart disease, ischemic stroke and a 1.5 to 3.6-fold increase in mortality [1]. T2DM is also a major risk factor for heart failure, peripheral arterial insufficiency and microvascular complications, affecting life quality and expectancy. It is estimated that, in general, patients with diabetes have a reduction in life expectancy of about 4–8 years, compared with individuals without diabetes [2].

The 2016 Global World Health Organization (WHO) Report of Diabetes estimated a worldwide adult diabetes prevalence in 422 millions of individuals in 2014, rising from 4.7% in 1980 to 8.5% in 2014, with the greatest increment in middle and low-income countries [3]. This number will probably overcome the previous WHO projection of 439 million adults with diabetes for 2030 [4]. Currently, 1.5 million deaths are directly attributed to diabetes each year. Although great advances in cardiovascular therapy and prevention have promoted outstanding reductions in diabetes-related coronary mortality in developed countries [5], cardiovascular morbidity and mortality still remain high in the majority of patients with diabetes. Considering the increasing number of cardiovascular event survivors and the global epidemic of T2DM, it is expected the number of patients with T2DM at a higher cardiovascular risk to rise, posing a giant challenge for health care systems worldwide. Cost-effective policies for reducing cardiovascular risk in this population are therefore urgently needed [6]. The present review focuses on the impact of risk factors in the global cardiovascular risk, and the role of detecting subclinical atherosclerosis and silent ischemia in the asymptomatic patient with diabetes.

## Cardiovascular risk in patients with diabetes

Diabetes has long been considered a “cardiovascular risk equivalent”. This statement was formerly based in the Finnish study [7], in which T2DM patients without coronary heart disease (CHD) events showed a similar coronary mortality as non-diabetic patients who had a previous coronary event. Diabetes also increases coronary death rates conferring the patient a worst prognosis after having the first CHD event [8]. These arguments led the 2001 NCEP-ATP III [9] to recommend patients with diabetes to be treated as a separated high-risk category, with no need for stratification.

Recent evidences indicate that CHD risk in T2DM is not universally similar to the risk of patients with prior cardiovascular disease, but is highly heterogeneous. A meta-analysis of 13 epidemiological studies, including 45,108 patients with and without diabetes observed that, in T2DM with no previous CHD, the CHD risk was 43% lower than when compared to individuals without diabetes with a prior myocardial infarction [10]. In a large population-based cohort [11] including 1,586,061 adults at ages 30–90 years, who were followed up for 10 years, the CHD risk was much lower among T2DM without CHD than in patients with CHD without diabetes: HR: 1.70 (95% CI 1.66–1.74) vs. 2.80 (95% CI 2.70–2.85). In another meta-analysis of observational studies with T2DM patients [12], cardiovascular risk was evaluated through coronary artery calcium score (CAC) at baseline. The authors found a 28.5% prevalence of patients with zero CAC scores, indicating a similar 5-year survival rate as in patients without diabetes [13]. Thus it is likely that a subgroup with lower CHD risk exists in T2DM, especially patients under 40 years old with shorter duration of disease.

Currently, the 2013 ACC/AHA guidelines [14], the 2016 ADA standards of diabetes care [15], the Brazilian Diabetes Society guidelines [16] and the 2016 European Society of Cardiology (ESC) [17] no longer consider diabetes as a coronary risk equivalent. The latest 2013 ACC/AHA guidelines [14, 18] now recommends stratification for patients with diabetes, when ages are from 40 to 75 years old, into two risk categories, using a global risk score calculator [14]. The recent ESC guideline considers that diabetes risk approaches the CHD risk when patients have more than 10 years of disease or when in the presence of renal dysfunction or microalbuminuria [17]. Patients younger than 40 years with a shorter duration of diabetes are currently defined as being part of a lower risk category. The categorization of diabetics into different cardiovascular risk groups by this way allows recognition of those who might benefit more from more intensive cardiovascular prevention. In the case of low-dose aspirin for example, considering the potential risk of gastrointestinal bleeding, defining cardiovascular risk by using a global risk score, might help guiding aspirin use in those with a greater net benefit.

Therefore, it might be useful to develop rationale strategies for detecting and treating more intensively patients at higher risk while it may be reasonable and cost-effective to use moderate therapies in those at lower cardiovascular risk.

## How can we stratify cardiovascular risk in diabetes?

### The American Heart Association and American College of Cardiology approach

The 2013 AHA/ACC guidelines [14] propose an approach based on the global risk estimation. The panel considers that patients with diabetes, either type 1 or 2, aged between 40 and 75 years, who have a baseline non-treated LDL-c between 70 and 189 mg/dL, should be stratified into a higher or a lower risk category to receive either high or moderate intensity treatment with statins. This categorization is based on the calculation of atherosclerotic cardiovascular disease (ASCVD) outcomes.

The ACC/AHA calculator was developed to estimate the 10-year risk for the first ASCVD event (non-fatal myocardial infarction or CHD death, or fatal/non-fatal stroke) by entering definite risk factors. It uses a risk equation derived from a pool of 4 cohorts obtained from the American population. The 10-year risk is based on the first ASCVD event over a 10-year period among people previously free from ASCVD. The calculator should be used in non-Hispanic African Americans and in non-Hispanic whites in the range of 40–79 years of age. For patients with diabetes, the guidelines indicate intensive statin treatment for patients with an ASCVD risk above 7.5% in 10 years. If the risk is below this cut-off, moderate-intensity statin treatment is indicated.

The panel defines high-intensity treatment as the statin therapy able to reduce LDL-c in more than 50% from baseline. Moderate-intensity treatment is defined as the reduction of LDL-c in about 30 to <50%. In patients aged less than 40 years, or above 75 years, the evidence of benefit is less clear. In these cases, when deciding to initiate or intensify statin therapy, the AHA/ACC panel consider reasonable to evaluate the potential for ASCVD benefits, considering the preferences of the patient and the potential for adverse effects.

### The American Diabetes Association approach

The 2016 American Diabetes Association Standards of Diabetes Care [15] endorses th**e** use of a risk factor-based approach to decide on initiation of statin therapy. Basically, it recommends risk stratification including 3 variables: age, the existence of previous cardiovascular events and the presence or not of risk factors. ADA risk factors include: LDL-c above 100 mg/dL, high blood pressure, smoking, overweight/obesity and family history of premature ASCVD. All patients with definite ASCVD events should receive high-intensity statin therapy, independently of age. In patients between 40 and 75 years without ASCVD events, but in the presence of cardiovascular risk factors, it is recommended high-intensity statin therapy. In older or younger patients, if in the presence of risk factors, either moderate or high-intensity statin therapy can be indicated, depending on the individual patient preference and tolerance. In older patients without ASCVD events and without risk factors, moderate statin therapy is advocated. In younger patients without ASCVD or risk factors, ADA considers that lifestyle therapy alone may be more appropriate. Recently, ADA has included a recommendation for patients with recent acute coronary syndrome. In these patients, generally high-intensity statins is appropriate, however, considering the results of the IMPROVE-IT study [19], ezetimibe plus moderate-statin therapy is now also advocated for those with LDL-c above 50 mg/dL, who cannot tolerate high-doses of statin.

## How to identify the high risk patient?

### Assessment of risk factors

#### Age

Age is the strongest non-modifiable risk factor for CVD. The increase in cardiovascular risk is continuous and progressive either in men or women. However, a transition to a high-risk category for developing cardiovascular disease seems to occur at a definite age for each gender. A large population-based retrospective cohort study [20], including 379,003 patients with diabetes and 9,018,082 adults without diabetes tried to define the age of transition from moderate to a high-risk condition in patients with diabetes. Considering a risk estimated above 20% in 10 years for the composite outcome of myocardial infarction, stroke and death from any cause, the transition to a high-risk category ocurred at 48 years in men and 54 years in women. When a broader definition of cardiovascular disease included revascularization, the age of transition fell to 41 and 48 years for both men and women, respectively. The transition from low to moderate risk category occurred at 35 and 45 years for both men and women considering the broader definition. Thus, for a person with diabetes to be considered at low risk, which means less than 10% risk estimate in 10 years, based only in age, they should be under 35 and 45 years respectively for men and women, provided that no other risk factor or evidence of cardiovascular disease is present. Thus the greatest efforts in reducing events should be focused in patients above these age limits.

#### Gender

In general population, the incidence of a new myocardial infarction is higher in men than in women, with an age-adjusted Hazard Ratio (95% CI) of 2.56 (2.53–2.60) [20]. In patients with diabetes, the men to women relation is much more narrow, however still higher in men: HR 1.22 (95% CI 1.18–1.25). Thus, the likelihood of a patient with diabetes to have an acute myocardial infarction solely due to gender is greatly attenuated when compared with individuals without diabetes [20].

When considering the mortality rate from coronary causes, women with diabetes are at a higher risk than men. In a meta-analysis of 37 studies [21] (including 447.064 T2DM patients), the relative risk (RR 95% CI) for fatal CHD between patients with and without diabetes was greater among women 3.50 (2.0–4.53), than in men 2.06 (1.81–2.34). Thus, in women with diabetes, the relative risk for a fatal coronary event is 50% higher than in men. This was probably explained by a less favorable cardiovascular risk profile in women linked to hypertension and hyperlipemia. The presumed reduced likelihood of women receiving the standard treatment for acute coronary syndrome and cardiovascular prevention is also important. Thus, women with diabetes seem to loose the protection due to gender greatly increasing the incidence of CHD in relation to men and with a greater mortality due to AMI.

#### Family history of coronary heart disease

The association of family history of myocardial infarction and incident coronary heart disease (CHD) in patients with diabetes is important, although the strength differs between studies. There are 2 large studies examining this association. The Women’s Health Study was a prospective cohort including 2642 postmenopausal women with diabetes without CHD at baseline followed up to 7 years with a 14.3% incidence of CHD. Compared with patients with diabetes without family history of CHD, the incidence of CHD in those with at least 1 first-degree relative was 50% higher (HR = 1.50 95% CI 1.20–1.87 p = 0.0003). In those who had 2 or more affected first-degree relatives, the incidence of CHD was 79% higher HR = 1.79 95% CI 1.36–2.35 p = 0.0001. The survival function was affected by both the number of relatives with CAD (p = 0.0002) as well as when CAD was considered premature (p = 0.004). Importantly, this study was fully adjusted for many covariates including systolic and diastolic blood pressure, smoking, race, lipid medication, physical activity and others [22].

In the MESA study [23], family history of fatal or non-fatal CHD in parents, siblings and children was considered an independent risk factor and performed better than Ankle Brachial Index, C-reactive protein and Flow Mediated Dilation. In a systematic review, family history of premature CHD was predictive for CHD, even when controlled for traditional risk factors. However, the addition of family history into a traditional risk factor model did not improve the discrimination [18]. The AHA/ACC 2013 guidelines [18] recommend to consider family history of premature coronary heart disease as a major risk factor, defined as male <55 years and female <65 years in any first degree relative.

#### Smoking

Cigarette smoking is one of the most important reversible risk factors for CHD. Compared with subjects who never smoked, the incidence of acute myocardial infarction is increased sixfold in women and threefold in men who smoke at least 20 cigarettes per day [24]. In a meta-analysis of 46 studies, including 130,000 patients with diabetes, the relative risk (95% CI) of smokers compared to non-smokers was 1.48 (1.34–1.64) for total mortality, 1.36 (1.22–1.52) for CV mortality, 1.54 (1.31–1.82) for CHD events, 1.44 (1.28–1.61) for stroke and 1.52 (1.25–1.83) for AMI [25].

Active smoking is associated with the highest risk of total mortality and cardiovascular events among patients with diabetes, while smoking cessation is associated with a reduced risk in total mortality and cardiovascular events in patients with diabetes. A large meta-analysis [26], (including 89 cohort studies of patients with diabetes) evaluated the effect of active smoking in mortality. Comparing patients who were active smokers with former smokers and never-smokers, active smoking was associated with more than 50% increase in mortality and CV events in comparison to non-smokers. However, former smokers were in higher risk of mortality and CVD events than “never-smokers”. Thus, there is an important benefit in smoking cessation among patients with diabetes, but a significant residual risk, which seems to be proportional to the exposed time of smoking, supports the concept that smoking should be suspended as early as possible.

#### Hypertension

Hypertension is a well-established risk factor for CHD and for stroke mortality. Isolated systolic hypertension is a major CHD risk factor at all ages, both in men and women [27]. In the Framingham study [28], diastolic blood pressure was the strongest predictor of CHD risk in patients under 50 years of age. In those with age between 50 and 59 years, all blood pressure parameters were predictors of CHD risk, while in those above 60 years of age, pulse pressure was the strongest predictor.

In both types 1 and 2 diabetes, hypertension is a major risk factor for ASCVD events and microvascular complications. In type 1 diabetes, hypertension is often the result of underlying diabetic kidney disease. In type 2 diabetes, it usually coexists with other cardiometabolic risk factors [15]. A recent meta-analysis [29] including 40 trials, and 100,354 adults with T2DM, evaluated systolic blood pressure (SBP) lowering. They observed that for each 10-mmHg lowering in SBP there was a significant lowering in risk for many outcomes such as: mortality (RR: 0.87; 95% CI 0.78–0.96); cardiovascular events (RR: 0.89 [95% CI 0.83–0.95], coronary heart disease (RR: 0.88 [95% CI 0.80–0.98]) and stroke (RR, 0.73 [95% CI 0.64–0.83]). The ADA 2016 standards of care recommends to treat people with diabetes and hypertension to a systolic blood pressure goal of 140 mmHg and a diastolic blood pressure goal of 90 mmHg [15].

#### Blood lipids

LDL-c is one of the most important reversible risk factors for cardiovascular morbidity and mortality. Cardiovascular (CVD) mortality data, from the ancillary observational MRFIT study [30], in the pre statin era, showed that, among 342,815 middle aged men in USA, (in which 5163 had diabetes) who were followed up for 16 years, the absolute adjusted risk of CVD death, stratified by cholesterol level, was several times higher in diabetics than in non-diabetics. The increase in CVD mortality tended to be disproportionately greater in patients with diabetes. The absolute excess risk due to diabetes ranged from 47.9/10,000 persons-years with total cholesterol <180 mg/dl to 103.8/10,000 persons-years for diabetic men in the 260–279 mg/dL total cholesterol range. The relative risk of CVD mortality for patients with diabetes ranged from 2.83 to 4.46 according to the level of cholesterol. Thus, cholesterol is a strong and independent risk factor for CVD mortality, which is potentiated by diabetes.

Considering reductions of LDL-c with statins, the Cholesterol Treatment Trialists’ (CTT) Collaborators meta-analysis [31] indicates that reducing LDL-c by 1 mmol/l with a statin will reduce the CVD relative risk in one-fifth, a linear phenomenon that is likely to occur similarly at any level of baseline LDL-c, at least through a limit down to LDL-c 50 mg/dL. In patients with diabetes, statins promote a proportional reduction of 9% in all-cause mortality (p = 0.02) and of 21% in incidence of major vascular events (p < 0.0001) per each mmol/l of reduction in LDL-c. Besides that there are also significant reductions in acute myocardial infarction (AMI) (p < 0.0001), coronary revascularization (p < 0.0001) and stroke (p < 0.0002).

## Assessment through risk score calculators

Risk calculators estimate the global cardiovascular risk based on the weight of independent risk factors in a mathematical equation, which generates a score based in absolute risk for different outcomes. This enables the clinician to estimate an individual patient’s risk to decide therapy. There are currently, at least 110 different cardiovascular risk score calculators and 45 exclusively for patients with diabetes [32]. Due to differences in databases, different combinations of CVD endpoints and in diverse mathematical algorithms, there is a considerable variability. Importantly, we should remind that the validation of these scores is limited to the characteristics of the population studied.

The UKPDS risk engine [33] was originally designed by the Oxford University and may be the most popular global risk calculator for patients with diabetes. It stratifies risk in patients with diabetes based mainly on the risk for coronary heart disease (CHD) in ten years. The database was derived from the UKPDS study cohort, a multi-ethnic population that included patients from UK, Greece, Spain and China. Components include age, duration of diabetes, gender, systolic blood pressure, total cholesterol, HDL cholesterol, smoking status, ethnicity and atrial fibrillation. The main outcome is the 10-year CHD incidence. When above 15%, it has a good sensitivity: 89.8% (95 CI 82.0–95.0) but with a low specificity: 30.3% (95% CI 25.4–35.6). In general, there is an overestimation of 108.8% in men and in 51.3% in women [33]. The UKPDS-RE calculator version 2 was recommended by 2014 Brazilian Diabetes Society to stratify risk in patients with diabetes [16], but will be replaced by risk factor stratification in the current (2017) update (data unpublished). UKPDS-RE may be useful in some situations where evidence-based decisions are lacking. Particularly it may help decisions in lower risk patients, those younger than 40 years old with or without risk factors, although it may have limitations in accuracy and may be time-consuming.

## High risk conditions associated with diabetes

### Long duration and early diagnosis of diabetes

Duration of diabetes is a key determinant of cardiovascular and CHD risk in diabetes. Patients with diabetes duration longer than 10 years can be considered in particular increased risk [11]. In a prospective observational study [34], with the aim to assess the incidence of coronary heart disease events and cardiovascular mortality according to the time of T2DM diagnosis, 4045 men, aged 60–79 years, were followed up for a mean of 9 years and classified at the entry into 4 groups: (1) no AMI/no T2DM, (2) no AMI/late-onset T2DM (diagnosis after age of 60 years), (3) no AMI/early-onset T2DM (before age of 60) and (4) prior AMI/no T2DM. Patients with both AMI and T2DM were excluded. There were a total of 372 major CHD events and 455 deaths. Compared to non-diabetic individuals, T2DM had a greater mean risk for CHD events and mortality, however, only patients with T2DM diagnosed before age of 60 with a mean duration of 16.7 years showed similar CHD risk as those with previous MI without diabetes. The adjusted hazard ratios (95% CI) for conventional risk factors and novel risk markers in relation to group 1 were: 1.54 (1.07–2.21), 2.39 (1.41–4.05), and 2.51 (1.88–3.36), for groups 2, 3 and 4, respectively.

Although age of onset and duration of diabetes are interrelated, the diagnosis of diabetes at an early age may confer an additional risk independently of diabetes duration. In a large cross-sectional survey [35], using data from the China National HbA1c Surveillance System (CNHSS), 222,773 patients with T2DM were divided into 2 groups according to the beginning of diabetes: (1) at early onset (mean 35 years of age) and (2) at late onset (mean 55 years of age). Both groups were then compared for non-fatal CV events. The odds-ratio (95% CI) adjusted for the duration of diabetes was: OR 1.91 (1.81–2.02), with the higher risk in the group with earlier onset of T2DM. Thus it may be possible that patients with earlier onset T2DM might have a more vulnerable phenotype due to obesity and low socio-economic [36].

### Low glomerular filtration rate and microalbuminuria

Glomerular filtration rate (GFR) is an independent risk factor for the development and severity of coronary artery disease [37]. Both decreased glomerular filtration rate and proteinuria independently increase the cardiovascular risk [38, 39]. A meta-analysis of cohort studies, including 105,872 individuals from the general population, with measurements of urine albumin-to-creatinine ratio (ACR) and 1,128,319 with urine protein dipstik, all-cause mortality was compared during a mean of 7.9 years of follow up. In 7.9 years of follow-up, the all-cause mortality for GFRs 60, 45, and 15 ml/min/1.73 m^2^ were respectively: HR [95% CI] 1.18 (1.05–1.32), 1.57 (1.39–1.78) and 3.14 (2.39–4.13).

Microalbuminuria is an independent risk factor for mortality both in healthy [40] and T2DM patients [41]. It is also an index of increased cardiovascular vulnerability. In the HOPE study [42], microalbuminuria was detected at baseline in 32.6% of T2DM patients and in 14.8% of non-DM patients. After 4.5 years of follow up, the relative risk (95% CI) for the primary end point (myocardial infarction, stroke or CV death) in T2DM was 1.97 (1.68–2.31) and in non-DM: 1.61 (1.36–1.90). The adjusted relative risk for major CV events was 1.83 (1.64–2.05) and for all cause of deaths: 2.09 [1.84–2.38]. For every 0.4 mg/mmol of increase in albumin-to-creatinine ratio, the adjusted hazard ratio of major CV events increased by 5.9% [95% CI 4.9–7.0%].

Despite a clear association between GFR and microalbuminuria with cardiovascular outcomes, data on reclassification, discrimination, calibration and cost-effectiveness are not available for considering practical recommendation of these markers for risk stratification. By this way, the AHA 2013 guidelines do not recommend its use in risk stratification either in diabetes or in general population [18].

### Presence of metabolic syndrome

The impact of metabolic syndrome (MetS) in cardiovascular risk stratification has long been discussed. MetS is associated with a twofold increase in cardiovascular outcomes and a 1.5-fold increase in all causes mortality. In a large meta-analysis [43], including 951,083 patients, the relative risk (95% CI) for cardiovascular outcomes were: for CVD: RR 2.35 (2.02–2.73), for CVD mortality: RR: 2.40; (1.87–3.08), for all-cause mortality: RR: 1.58; (1.39–1.78), for myocardial infarction: RR: 1.99; (1.61–2.46) and for stroke: RR: 2.27; (1.80–2.85).

The still open question regarding MetS is whether MetS is simply the result of the sum of their components or if there is an independent excess of risk. Based on the NHANES III data [44], a large cross-sectional populational study including 76.1 million of US individuals, MetS was present in 44% of the total population above 50 years old and in 87% of patients with T2DM. In this study, there were 13,013,000 individuals with diabetes. The total population was divided into 4 categories, according to the presence of diabetes and MetS. In patients with both DM and MetS, the prevalence of CHD was 19.2%, while in patients with DM without MetS the CHD prevalence was 7.5%. In individuals without DM, with or without MetS, had a CHD prevalence respectively of 8.7% to 13.9%. Despite being a good predictor of CHD in the univariate analysis, MetS was not a significant predictor in the multivariate analysis, when it was adjusted for triglycerides, HDL-c, blood pressure, fasting glucose and diabetes. So the risk impact of MetS seems to be derived from its individual components, especially HDL-c and blood pressure. However, there was an increase in the attributable risk of MetS when diabetes was present, rising from 37.4% to 54.7%. Most importantly, independently from the origin of MetS risk, the prevalence of CHD markedly increases with presence of MetS, especially in the presence of diabetes.

### Chronic hyperglycemia

In individuals without diabetes, fasting blood glucose (FPG) has a curvilinear association with vascular disease and it is considered a moderate risk factor [1]. In a meta-analysis of 102 prospective studies [1], 698,782 individuals from different strata of FPG were compared with patients with normal FPG (70–100 mg/dL). The HR (95% CI) for coronary heart disease in patients in the group with mean FPG of 100–110 mg/dL and FPG 110–126 mg/dL were respectively: 1.11 (1.04–1.18) and 1.17 (1;08–1.26), showing only a modest rise. However, when patients with new diabetes were compared (FPG > 126 mg/dL) the HR rose to 1.78 (1.56–2.03) and was still higher when compared to patients with known diabetes: 2.36 (2.02–2.76).

### Severe hypoglycemia

Severe hypoglycemia (SH) (defined as hypoglycemic episode requiring assistance) increases approximately twice the risk of cardiovascular disease in T2DM [44]. A cohort study [45] followed 906 T2DM patients with ages 25–75 years for a median of 10.4 years, looking for an association between severe hypoglycemia and the primary outcome (death of any cause or CV death). The authors observed that, after adjusting for many covariates, severe hypoglycemia was still strongly associated with an increase in all cause mortality [HR 2.64 (1.39–5.02) p = 0.003] and CV mortality HR 6.34 (2.02–19.87 p = 0.002). In this study, patients who experienced severe hypoglycemia had 2.64 times higher risk of all-cause mortality compared with those without severe hypoglycemia episodes. In the Hong Kong Diabetes Registry study [46], patients with severe hypoglycemia also showed increased incidence of mortality compared with those without SH, respectively: (32.8 vs. 11.2% p < 0.0001). A plausible mechanism in which hypoglycemia may be linked to cardiovascular events is the acute induction of pro-inflammatory and pro-atherosclerotic mediators. In an experiment using euglycemic and hypoglycemic clamp in healthy individuals and type 1 diabetes patients [46], moderate hypoglycemia acutely increased circulating levels of PAI-1, VEGF, vascular adhesion molecules (VCAM, ICAM, E-selectin), IL-6, and markers of platelet activation (P-selectin) in individuals with type 1 diabetes and in healthy individuals.

### Non-alcoholic fatty liver disease

Non-alcoholic fatty liver disease is an independent predictor of CAD among patients with T2DM. The Valpolicella Heart Diabetes Study [47], a prospective nested case–control study,  was designed to evaluate the association between non-alcoholic fatty liver disease, detected by ultra-sonography, and the incidence of cardiovascular complications in T2DM. The study included 2103 individuals with T2DM free of CVD at baseline who were followed for 6.5 years. There were 384 cases of new CVD events. After multiple adjustments for sex, age, smoking, diabetes duration, A1C, LDL-c, medications (hypoglycemic, antihypertensive, lipid-lowering, antiplatelet drugs) and metabolic syndrome, the association remained independent (HR 1.87 [1.2–2.6], p < 0.001). NAFLD has also been associated to diastolic dysfunction in T2DM patients without a history of ischemic heart disease [48] and with increased coronary calcium scores, independently of features of metabolic syndrome [49].

### Obstructive sleep apnea (OSA)

Obstructive sleep apnea is characterized by recurrent episodes of partial or complete upper airway collapse and obstruction during sleep, associated with intermittent oxygen desaturation, sleep fragmentation and is associated with an increased incidence of fatal myocardial infarction and stroke [50, 51]. The prevalence of OSA is increased in T2DM patients and could be underdiagnosed. In one series from Germany [52], 938 T2DM men, who answered a questionnaire, 56% were at increased risk for OSA, and diabetes was an independent predictor for OSA. Prevalence of OSA is also increased in T1DM patients [53]. Severe OSA (apnea-hypopnea index [AHI] >30/h) is strongly associated with increased mortality, stroke and cardiovascular disease in middle-aged populations, on the other hand, patients with diabetes are at high risk of OSA and should be questioned for symptoms, which may warrant further investigation and treatment [54].

### Erectile dysfunction

Men with erectile dysfunction (ED) are at higher risk for cardiovascular (CV) events [55]. In a metanalysis of cohort and cross-sectional studies [56] including 22,586 subjects with T2DM and 3791 CV events, the overall odds ratio (OR) of diabetic men with ED compared with patients without ED, was 1.74 (95% CI 1.34–2.27; p = 0.001) for CV events and 1.72 (95% CI 1.5–1.98; p, 0.001) for CHD, considering only the cohort studies. In the cross-sectional studies, the OR of DM men with or without ED was 3.39 (95% CI 2.58–4.44; p, 0.001) for CV events and 3.43 (95% CI 2.46–4.77; p < 0.001) for CHD events. Thus, ED may precede CVD in the same disease progression line, and its presence in a patient with diabetes indicates a higher CV risk condition.

## Role of biomarkers in patients with diabetes

The assessment of the utility of risk biomarkers involves several statistical tests beyond the statistical association [57, 58]. Indeed, the presence of a statistically significant association between a risk marker and the disease is mandatory, but does not guarantee the improvement in risk prediction. Recent literature has proposed the use of measures of discrimination and calibration to test the prediction capacity of a risk marker. Discrimination is the capacity to identificate the subject who will present the event of interest from the one who will not. The area under the receiver-operating-characteristic (ROC) curve (AUC) is a popular metric of discrimination. The AUC represents the area under the plot of sensitivity (true positive rate) versus one minus specificity (true negative rate), and means the probability that a given test or predictive model assigns a higher probability of an event to those who actually develop the event [57, 58]. Due to the poor performance of various risk markers on their ability to increase the AUC, researchers have also tested other approaches to analyse the predictive utility of a marker, such as the reclassification. The reclassification evaluates the capacity of a new test when added to a model, to properly reassign a subject to a higher or lower category of risk [57, 58]. Calibration is another statistical method to test the predictive capacity of a marker and represents a measure of how close the predictive risks by a certain model are of the real risks, after dividing the population into categories, such as deciles.

### High sensitivity C-reactive protein

High sensitivity C-reative protein (hs-CRP) is a marker of systemic inflammation and a predictor of incident CVD and CHD, independent of diabetes. In the study by Ridker et al. [59], 27,939 presumed healthy American women were followed up for a mean of 8 years for incident myocardial infarction, ischemic stroke, coronary revascularization or death from cardiovascular causes. They observed that hs-CRP was strongly related to the incidence of cardiovascular events, even after adjustments for age, smoking status, diabetes, categorical levels of blood pressure and the use of hormone therapy. In that study, hs-CRP performed better than LDL-c, indicating that hs-CRP may add substantial prognostic information to that conveyed by the Framingham risk score. The MESA study [23], confirmed that hs-CRP is independently associated with incident CHD, adding information to traditional risk factors of Framingham risk score.

In patients with diabetes, the predictive value of CRP for cardiovascular disease is a more debated issue. In a pooled analyses of 25,979 participants from 4 UK prospective cohort studies [60] followed for a median of 93 months, CRP was associated with a 53% (95% CI 43–64) and 43% [37–48] increase in cardiovascular risk and all-cause mortality. In individuals with diabetes, CRP was associated with 54% increase in cardiovascular death and 53% greater risk of all cause mortality. Across subgroups of participants based on 3 categories of CRP (<1, 1–3, and >3 mg/l), there was a graded association between CRP and outcomes, except in people with diabetes. Similar results were also seen in the Hoorn study [61], in the Strong Heart Study [62], in the Honolulu Heart Program study [63] and in the study by Biasucci et al. [64]. In these studies, CRP was a significant predictor of CVD only among participants without diabetes. Interestingly, in the Diabetes Heart Study [65], hs-CRP was evaluated for predicting mortality in 846 T2DM who were followed up for a period of 7.3 years. Baseline hs-CRP values were compared in living and in deceased sub-groups. Baseline CRP was significantly higher in the deceased sub-group (9.37 ± 15.94) compared with the living subgroup (5.36 ± 7.91 mg/l; p < 0.0001). A hs-CRP above 10 mg/l indicated an Odds Ratio of 5.24 (2.80–9.38) to be deceased. Although a retrospective study, it indicates that CRP may predict mortality in T2DM, but at a higher value than the American Heart Association CRP threshold of >3 mg/l.

## Other biomarkers

In a prespecified subgroup analysis of Saxagliptin Assessment of Vascular Outcomes Recorded in Patients with Diabetes Mellitus (SAVOR)-Thrombolysis in Myocardial Infarction (TIMI) 53 trial, there was an evaluation of biomarkers reflecting the pathophysiologic processes of myocardial injury with high-sensitivity troponin T (hsTnT), hemodynamic stress with N-terminal pro–B-type natriuretic peptide (NT-proBNP), and inflammation with hsCRP, to assess their incremental prognostic value in the risk stratification of diabetics [66]. In this secondary analysis of a study including over 12,000 patients with established cardiovascular disease or multiple risk factors, elevated levels of hsTnT, NT-proBNP, and hsCRP improved discrimination and correct reclassification of the risk for the primary endpoint (cardiovascular death, myocardial infarction, or ischemic stroke), the individual endpoints, and hospitalization for heart failure [66].

## Assessment of subclinical atherosclerosis

### Coronary artery calcium score (CAC)

Coronary arterial calcification is part of the development of atherosclerosis, occurring almost exclusively in atherosclerotic arteries being absent in normal vessel walls [67]. Atherosclerotic plaque proceeds through progressive stages where instability and rupture can be followed by calcification, providing stability to an unstable lesion [68]. Coronary artery calcium score (CAC) is determined by electron-beam (EBCT) and multi-detector (MDCT) computed tomography [69]. It has a strong correlation with the total coronary atherosclerotic burden and is able to define CHD risk, being an independent predictor of cardiovascular disease [70, 71].

The MESA Study [23] compared the improvement in prediction of incident CHD and CVD between six risk markers in 6814 patients, in whom 1330 were at intermediate risk, determined by the Framingham risk score (FRS) at baseline. After a 7.6-year median follow up, there were 94 CHD and 123 CVD events. Baseline accuracy ROC curve for CAC afforded the highest increment of sensitivity and specificity to the FRS compared to all other markers. CAC score has an incremental relationship with higher event rates when compared to a CAC zero score. A metanalysis [70] evaluating the prognostic value of CAC from 4 studies in asymptomatic subjects, indicated a linear relationship between CAC and CHD events. The summary adjusted relative risk ratios for scores ranging from CAC 1–100 compared to CAC zero was 2.1 (95% CI 1.6–2.9). The RR for CAC 101–400 and CAC >400 compared to CAC zero ranged from 3.0 to 17.0, but varied significantly among studies.

In patients with diabetes, cross-sectional studies have shown higher prevalences and extent of coronary calcium compared with non-diabetic patients, with a great heterogeneity [71–73]. In one series, of 155 asymptomatic individuals with diabetes, 72% had positive CAC scores and 48% had a CAC score >102^72^.

Coronary arterial calcification is predictive for cardiovascular end-points in asymptomatic patient with T2DM. The PREDICT study [74], aimed to evaluate the CAC score as a predictor of cardiovascular events in type 2 diabetes. They included 589 T2DM patients with no history of cardiovascular disease, mean age 63 years, predominantly overweight male, who had CAC score measured at baseline. Patients were followed for a median of 4 years for cardiovascular endpoints. CAC was a highly significant independent predictor of events (p < 0.001). A doubling in CAC was associated with a 32% increase in risk of events. There was a progressive increase in hazard ratio according with the CAC score level, comparing to CAC <10. Moreover, the area under the ROC curve of Framingham risk score and UKPDS-Risk Engine increased significantly with the addition of CAC score.

Coronary arterial calcification is also predictive for mortality in asymptomatic T2DM. In a large cohort study with a mean follow-up of 5 years [13], including 10,377 asymptomatic individuals, with 903 T2DM, the mean CAC score for individuals with and without diabetes were respectively 281 ± 567 and 119 ± 341 Agatston units (p < 0.0001). The death rate was 3.5% in T2DM and 2% in non-DM respectively (p < 0.0001). The increase in mortality was proportional to increases in CAC. Interestingly, the absence of coronary calcium (CAC = 0) conferred a similar survival rate for both groups with or without diabetes [72].

These associations were confirmed by a systematic review and metanalysis of 8 cohort studies [12] that investigated the association of CAC with all cause mortality and cardiovascular events in T2DM. The study included 6.521 T2DM patients with a mean follow up of 5.18 years. They compared the number of events in patients with CAC above and below 10. There were a total of 802 cardiovascular events. CAC below 10 was present in 28.5% of patients. The relative risk for all-cause mortality or CV events was 5.47 (95% CI 2.59–11.53 p < 0.001). In relation to the main outcome, a CAC score >10 presented respectively a sensitivity of 94% (95% CI 89–96) and a specificity of 34% (24–44%). Because people with a CAC < 10 were 6.8 times less likely to have a cardiovascular event, the authors suggest that the negative predictive value of CAC <10 may be useful to discriminate T2DM to a lower risk category.

The long-term predictive value of CAC score for all cause mortality in asymptomatic patients with diabetes was recently addressed in a 15-year cohort study [75]. Baseline CAC was determined in 9715 non-diabetic individuals and in 810 T2DM patients, predominantly male, with mean age of 53 years. In 34% of T2DM, baseline CAC score was zero (CAC = 0). The cumulative mortality rate over 15 years according to baseline CAC score was greater in T2DM than in non-diabetic individuals. The adjusted HR (95% CI) for mortality at 15 years was respectively: for CAC [0]: 2.53 (1.74–3.69); CAC [1–399]: 2.07 (1.64–2.62); CAC [>400]: 1.88 (1.41–2.51). Interestingly, a CAC zero conferred a similar mortality rate between T2DM and non-DM patients for the first 5 years. After 5 years, however, the risk of mortality increased significantly for diabetic patients even in the presence of a baseline CAC = 0 [75].

Although the importance of CAC in risk stratification has increased significantly in the last years, ADA 2016 [15] still does not recommend CAC score for routine use in risk stratification of patients with diabetes, due to still open questions in cost-effectiveness. Further studies should address this point specially taking into account the risks of excessive exposition to radiation and costs.

### Carotid intima-media thickness (CIMT) and carotid plaque

Carotid-wall intima-media thickness (CIMT) is the distance from the lumen-intima interface to the media-adventitia interface of the artery wall, determined by a carotid artery ultrasound [76]. CMIT is a surrogate marker for new acute myocardial infarction and stroke in individuals above 65 years old [77], when maximal IMT is above 1.11 mm, both in common and in internal carotid arteries. Increased CIMT above 1 mm is also predictive for CHD in younger individuals without previous cardiovascular events [78]. The addition of maximal CMIT measurement of the internal carotid to the Framingham Risk Score (FRS) only modestly (7.6% p < 0.001) improves its accuracy for predicting cardiovascular events [79]. In patients with T2DM, CMIT above 1.9 mm is predictive of coronary artery stenosis, improving the FRS and UKPDS risk engine scores accuracy in Japanese population [80]. Interestingly, CMIT seems to perform better in obese than in lean T2DM patients [81].

A carotid plaque is defined as the thickness of the intima above 1.0 mm [82], 1.1 mm [79] or even 1.5 mm [83]. The total plaque area determination is a simple and highly reproducible method to quantify atherosclerosis. It improves significantly the sensitivity of FRS as a screening tool to reclassify intermediate and high risk in non-diabetic patients [82]. In asymptomatic patients with T2DM, the sum of the maximum plaque thickness above 1.1 mm from both sides of the carotid wall carotid plaque, increases the predictive value for detecting coronary stenosis greater than 50% (obstructive CAD). This seems to be independent of age, hypertension, hyperlipidemia and HbA1c [83].

Although promising, CIMT and carotid plaque detection are currently not recommended for routine use for risk assessment by AHA/ACC 2013 guidelines [14, 18]. The panel considers that additional research is needed to quantify the cost effectiveness and the impact of imaging for subclinical atherosclerosis on cardiovascular risk factor management and patient outcomes.

### Ankle-braquial index (ABI)

Ankle-braquial index is obtained by measuring systolic blood pressure in the supine position, in bilateral brachial arteries, dorsalis pedis arteries and in posterior tibial arteries using Doppler with a 5-mHz probe. The highest value of each blood pressure measurement is used. Most of studies have used a cut-off point of <0.90 [84–87]. A low ABI score is associated with elevated cardiovascular risk. In the MESA study [23], ABI was compared to other cardiovascular risk markers in relation to Framingham risk score. ABI was superior to Framingham Score alone and was considered an independent risk predictor of incident CHD/CVD beyond traditional risk factors. In a systematic review including 9 studies, the sensitivity and specificity of a low ABI as a predictor of future CVD events were respectively 16.5% and 92.7% for coronary heart diseases, 16.0% and 92.2%, for incident stroke and 41.0% and 87.9% for cardiovascular mortality. Thus ABI has a high specificity but a very low sensitivity, limiting its utility as a screening test for CAD [86]. The ACC/AHA Expert Opinion guidelines [14, 18] currently recommend ABI threshold of <0.9 for considering a patient at high risk, in the cardiovascular risk assessment of asymptomatic adults at intermediate risk.

In patients with diabetes data is less available. In a Chinese study in T2DM patients, ABI <0.9 was independently associated with high risk for all-cause mortality and CVD mortality [88]. In that study, decreasing ABI scores below 0.9 presented a progressive association with mortality.

## Assessment of silent ischemia

Silent ischemia identification may be important for a more aggressive intervention to prevent clinical events or, in advanced disease, to indicate revascularization. In diabetes, silent coronary artery disease occurs in a varying prevalence, ranging from 12.4% [89], 18% [90], 22% [91] up to 34% [92], depending on age, duration of diabetes and presence of risk factors. Because symptomatic diabetic patients may have a worse prognosis, the detection of disease before acute coronary syndrome events may improve morbidity and mortality.

The 2016 position from the American Diabetes Association, however, does not recommend screening asymptomatic high-risk patients with diabetes, for not considering it cost-effective. There is a lack of evidence indicating that screening these patients could reduce cardiovascular outcomes. The DIAD study [93] assessed whether routine screening for CAD could identify asymptomatic T2DM patients and how screening with adenosine-stress myocardial perfusion imaging (sMPI) could affect their cardiac outcomes. In a randomized controlled trial, 1123 participants with T2DM and no symptoms of CAD were randomly assigned to be screened or not with sMPI. After a follow up of 4.8-years, no additional benefit was observed. There were 2.7% events among the screened group and 3.0% in the non-screened group, which was not significantly different [HR], 0.88 (95% CI 0.44–1.88).

Another point is that, there is evidence [94] that silent ischemia may attenuate with time. In the DIAD study [93] 56 (79%) of 71 T2DM patients who had positive sMPI demonstrated complete resolution of ischemia when the test was repeated 3 years later. The last point is that, the great majority of T2DM patients with silent ischemia would have less severe ischemia [93]. In these presumed cases, patients should benefit of intensive  prevention similarly as invasive revascularization treatment [95, 96]. Currently, AHA and ADA guidelines recommend only resting ECG for routine evaluation of asymptomatic patient with diabetes [15, 18].

## Conclusions

Type 2 Diabetes increases cardiovascular risk in 2 to fourfold, but cannot be considered a risk equivalent due to the high heterogeneity. Risk stratification is necessary to individualize treatment. It is expected that almost 30% of cases may have a 5-year CHD risk similar to general population, however, lifetime risk seems to be invariably high in almost all patients with diabetes. Age above 40 years, diabetes diagnosis of more than 10 years, the presence of a first degree family history with premature CHD, male gender, high blood pressure, LDL above 100 mg/dl, low renal function, microalbuminuria, presence of non-alcoholic fatty liver disease, obstructive sleep apnea, erectile dysfunction and specially metabolic syndrome, chronic hyperglycemia and severe hypoglycemia are conditions that increase cardiovascular risk.

For now, risk stratification in the patient with diabetes should include solely the traditional risk factors with or without risk calculators. Emerging risk factors are still awaiting confirmatory studies. Basically for being useful in clinical practice, first they must be strongly associated with the outcome. Secondly, there must be a reasonable potential for reclassification besides the traditional risk factors. They also must have good discrimination and calibration. Finally, they must have a favorable cost-effectiveness profile. Coronary artery calcium score, hs-CRP, family history of premature CVD and ABI can be useful tools. Estimated glomerular filtration rate (GFR) and microalbuminuria roles are still uncertain. CMIT is currently recommended against for using in clinical practice by 2013 AHA [18]. Better stratification of patients with diabetes may improve quality of indication of treatment in patients with diabetes.

## Abbreviations

ABI: ankle-brachial index
ACR: albumin-to-creatinine ratio
ADA: American Diabetes Association
A1C: hemoglobin A1C
AHA/ACC: American Heart Association/American College of Cardiology
AHI: apnea hypopnea index
AMI: acute myocardial infarct
ARR: absolute risk reduction
ASCVD: atherosclerotic cardiovascular disease
CAC: coronary artery calcium score
CHD: coronary heart disease
CMIT: carotid intima-media thickness
CNHSS: China National HbA1c Surveillance System
CI: confidence interval
CRP: C-reactive protein
CV: cardiovascular
CVD: cardiovascular disease
CKD: chronic kidney disease
EBCT: electron-beam computed tomography
ECG: eletrocardiogram
ED: erectile dysfunction
FMD: flow mediated dilation
FPG: fasting plasma glucose
FRS: framingham risk score
GFR: glomerular filtration rate
HDLc: high density lipoprotein
HR: hazard ratio
hs-CRP: high sensitivity c-reactive protein
IL-6: interleukin 6
ICAM: intercellular adhesion molecule
LDL-c: low density cholesterol
MDCT: multi-detector computed tomography
MetS: metabolic syndrome
MPI: adenosine-stress myocardial perfusion imaging
NAFLD: non-alcoholic fatty liver disease
NCEP-ATP III: National Cholesterol Education Program-Adult Treatment Panel III
NHANES III: National Health and Nutrition Examination Survey
OR: odds-ratio
OSA: sleep apnea
PAI-1: plasminogen activator inhibitor-1
ROC: receiver operating characteristic
RR: relative risk
SBP: systolic blood pressure
T1DM: type 1 diabetes
T2DM: type 2 diabetes
VEGF: vascular endothelial growth factor
UK: United Kingdom
UKPDS-RE: United Kingdom Prospective Diabetes Study Risk Engine
VCAM: vascular cell adhesion molecule
WHO: World health Organization
