# Erratum to: Cardiovascular risk assessment in patients with diabetes

**DOI:** 10.1186/s13098-017-0270-9

**Published:** 2017-09-19

**Authors:** Marcello Casaccia Bertoluci, Viviane Zorzanelli Rocha

**Affiliations:** 1 Departamento de Medicina Interna da Faculdade de Medicina da, Universidade Federal do Rio Grande do Sul, UFRGS, Rua Ramiro Barcelos 2400, Porto Alegre, ZIP 90035-003 RS Brazil; 2 Universidade Federal do Rio Grande do Sul, Programa de Pós-Graduação em Ciências Médicas, Rua Ramiro, Barcelos 2400, Porto Alegre, 90035-003 Brazil; 3 Hospital de Clínicas de Porto AlegreUFRGS, Serviço de Medicina Interna, Rua Ramiro Barcelos 2350 Sala 700, Porto Alegre, 90035-005 RS Brazil; 4 Lipid Clinic, Heart Institute (InCor) University of Sao Paulo, Sao Paulo, SP 05403-900 Brazil

## Erratum to: Diabetol Metab Syndr (2017) 9:25 DOI 10.1186/s13098-017-0225-1

Upon publication of the original article [1], it was noticed that the Ref. [32] was incorrectly given as [32]: Allan GM, Nouri F, Korownyk C, Kolber MR, Vandermeer B, McCormack J. Agreement among cardiovascular disease risk calculators. Circulation. 2013;127(19):1948–56.

The correct Ref. [32] is

32. van Dieren S, Beulens JWJ, Kengne AP, Peelen LM, Rutten GEHM, Woodwarth M, van der Schouw T, Moons KGM. Prediction models risk of cardiovascular disease in patients with type 2 diabetes: a systematic review. Heart. 2012;98:360–9. doi:10.1136/heartjnl.2011-30074.

 This has now been acknowledged and corrected in this erratum.

The sentence in the section ‘Assessment through risk score calculators’, “There are currently, at least 110 different cardiovascular risk score calculators and 45 exclusively for patients with diabetes [32]” is incorrectly given.

The correct sentence is

There are currently, at least 45 different cardiovascular risk score calculators and 12 exclusively for patients with diabetes [32].

This has now been acknowledged and corrected in this erratum.
